# Sleep Deprivation and Gut Microbiota Dysbiosis: Current Understandings and Implications

**DOI:** 10.3390/ijms24119603

**Published:** 2023-05-31

**Authors:** Jingyi Sun, Dan Fang, Zhiqiang Wang, Yuan Liu

**Affiliations:** 1 Jiangsu Co-Innovation Center for Prevention and Control of Important Animal Infectious Diseases and Zoonoses, College of Veterinary Medicine, Yangzhou University, Yangzhou 225009, China; 2 Joint International Research Laboratory of Agriculture and Agri-Product Safety, The Ministry of Education of China, Yangzhou University, Yangzhou 225009, China; 3 Institute of Comparative Medicine, Yangzhou University, Yangzhou 225009, China

**Keywords:** gut microbiota dysbiosis, immune system, metabolism, sleep deprivation

## Abstract

Gut microbiota comprises the microbial communities inhabiting our gastrointestinal (GI) tracts. Accordingly, these complex communities play a fundamental role in many host processes and are closely implicated in human health and diseases. Sleep deprivation (SD) has become increasingly common in modern society, partly owing to the rising pressure of work and the diversification of entertainment. It is well documented that sleep loss is a significant cause of various adverse outcomes on human health including immune-related and metabolic diseases. Furthermore, accumulating evidence suggests that gut microbiota dysbiosis is associated with these SD-induced human diseases. In this review, we summarize the gut microbiota dysbiosis caused by SD and the succedent diseases ranging from the immune system and metabolic system to various organs and highlight the critical roles of gut microbiota in these diseases. The implications and possible strategies to alleviate SD-related human diseases are also provided.

## 1. Introduction

Sleep, a pillar of life that takes up almost one-third of our daily time [1], is essential to our health. Evidence has proven that sleep plays a crucial role in brain functions including cognitive performance [2], memory consolidation [3,4], and mood regulation [5,6,7]. Moreover, sleep takes part in nearly all systems of the whole body: for example, the autonomic nervous system [8,9], cardiovascular system [8,10], immune system [11,12], and metabolism [13,14]. Unfortunately, sleep deprivation (SD), whose definition is the body’s incapacity to get enough sleep [15], has become commonplace in modern society. Previous research has found that 20% to one-quarter of the population of the United States is forced to face it [16,17,18] because of improper diet [19], pressure [20,21], and increasing brain stimulation by smartphones [1]. Possible adverse outcomes elicited by SD include depression (in a bidirectional relationship with SD) [22,23,24,25,26], obesity [27,28], diabetes [14,29], cardiovascular disease [30,31], cancer [32,33], neurological dysfunction [31,34], and even death [34]. Therefore, a better understanding of the association between SD and human diseases, as well as the underlying mechanisms of action, is of critical importance.

All higher animals have indiscerptible relationships with microorganism communities including bacteria, fungi, viruses, archaea, and protozoa [35,36]. Among these, up to 100 trillion microbial cells [36,37], 1000 to 1500 bacterial species assemble in the gastrointestinal tract, while an individual only contains about 160 species [38]. The 16S rRNA amplicon sequencing results have shown that Firmicutes and Bacteroidetes are the most abundant phyla [38,39]. Proteobacteria, Verrumicrobia, Actinobacteria, Fusobacteria, and Cyanobacteria also account for a relatively large proportion of the total population [37]. Such an enormous colony masters a considerable power, being able to tailor metabolism [40,41], the immune system [42,43], the gut-organ axis [44,45,46,47,48,49], and so on. Thus, gut microbiota is also termed as the second brain or a ‘forgotten organ’ [36,50]. When the gut microbiota’s balance between commensal and pathogenic microbiomes leans to the harmful side, or its homeostasis is disturbed by unhealthy lifestyles or various environmental factors, the whole body will suffer from a series of diseases such as inflammatory bowel disease (IBD) [51], obesity [52], and type 2 diabetes mellitus (T2DM) [53].

Recently, increasing studies have shown that there is a trend for the changes in gut microbiota elicited by SD to spread deleterious effects to other systems, rather than being limited in the gastrointestinal (GI) tract, because of the complicated interconnections between gut microbiota and their hosts [54,55]. In this review, we depict the consequences of gut microbiota dysbiosis caused by SD and elucidate the potential biological mechanisms. We also highlight the implications covering the possible ways to reverse the adverse effects of SD from two different perspectives.

## 2. The Broken Homeostasis of Gut Microbiota Caused by SD

The dysbiosis of gut microbiota brought by SD mainly manifests in their abundances and compositions. There are two indexes widely used in analysis: alpha (α) diversity and beta (β) diversity. Alpha (α) diversity presents the mean species diversity in sites or habitats at a local scale [56], and beta (β) diversity indicates the variation in community compositions [56]. By performing 16S rRNA gene sequencing on fecal samples, most of the existing studies have demonstrated that α diversity experiences a dramatical decrease in the SD group compared with the control group, while β diversity displays different outcomes. Details for each experiment are presented in Table 1.

**Figure 1:**
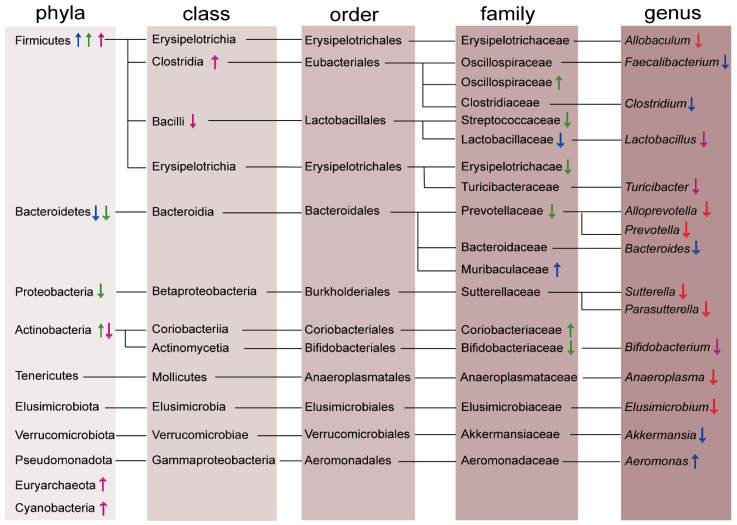
Gut microbiota alternation caused by SD. Taxonomic list of altered gut microbiota composition induced by SD. The shades of brown from light to dark represent the classification of phyla, classes, orders, families, and genera. Sleep losses of different durations are presented by the colors of the arrows: red—24 & 40 h SD, blue—3 days of SD, green—2 days of partial SD, and purple—repeated SD. The up/down arrows indicate an increase/decrease of the represented bacteria.

As for gut microbiota composition changes, since the SD duration differs from one experiment to another, the 16S profiling is differential. What makes common ground is that, in all reports, SD has caused a markable reduction of Bacteroidetes [54] and the microorganisms existing in the largest proportion—Firmicutes—have acquired a higher scale, with the ratio of Firmicutes to Bacteroidetes (F: B) rising up correspondingly. The complete summary of broken homeostasis in gut microbiota brought by SD is presented in Figure 1. Overall, SD depletes the number of specific beneficial bacteria such as probiotics that are intended to have health benefits when consumed or applied to the body, and increases the quantity of some pathogenic bacteria, like *g*_*Aeromonas*, that may cause diseases and illnesses [55].

## 3. Functional Impairment by SD and the Role of Gut Microbiota in This Process

In addition to causing direct changes in the gut microbiota, perturbations in these communities can also cause or exacerbate pathological changes in hosts. These derived changes demonstrate that gut dysbiosis plays a profound mediating role between SD and multiple diseases.

### 3.1. Weakened Immune Defenses or Colonization Resistance against Infections

#### 3.1.1. SD-Induced Depletion of Immune Defenses

**Figure 2:**
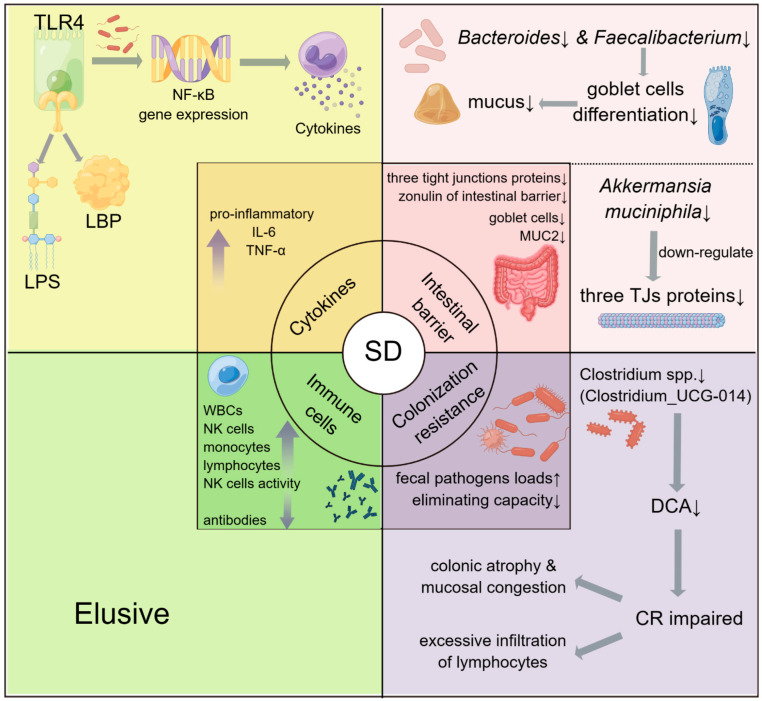
Summary of SD-induced immune impairments and the associations between SD and gut homeostasis. The inner circle and middle square concentrate on the symptoms of SD-induced diseases, covering aspects of cytokines [59], the intestinal barrier [62,63], immune cells [64,65], and colonization resistance [66]. The outer square demonstrates the mechanisms by which SD contributes to these diseases, especially those involved in gut microbiota. TLR4—Toll-like receptor 4; LPS—lipopolysaccharides; LBP—lipopolysaccharide-binding protein; NF-κB—nuclear factor kappa-B; WBCs—white blood cells; NK cells—natural killer cells; MUC2—mucin 2; TJs—tight junctions; DCA—deoxycholic acid; CR—colonization resistance. The up/down arrows indicate an increase/decrease of the represented object.

It has become increasingly evident that sleep and the immune system are closely connected. The immune system protects bodies from pathogen invasion and is divided into two parts determined by the speed and specificity of the immune response: innate and adaptive immunity. Accumulating studies have demonstrated that sleep loss can affect different parts of the immune system, resulting in a wide variety of disorders (Figure 2).

The first involved the cytokines, a series of protein molecules secreted by immune and other types of cells that signal other cells to regulate inflammation. In an experiment with 12 men and 13 women as subjects, the pro-inflammatory cytokines covering IL-6 and TNF-α at 24 h were remarkably increased after SD [59]. Similar results were also shown in other studies [26,60]. This change is involved in the upregulation of inflammatory reactions [61] and increases the risk for cardiovascular and metabolic disorders.

The second involved immune cell counts and functions. Immune cells are some of the significant parts of the immune system. They are developed from stem cells in the bone marrow and help the body fight against infections or other diseases. The counts of white blood cells (WBCs), monocytes, lymphocytes, and natural killer (NK) cells in subjects with SD all experienced concomitant increases [64,65,67]. Moreover, NK cell activity in particular was detected, and the results indicated that one night’s worth of total SD induced an acute and transient increase of NK cell activity in the experiment with ten healthy adult men as subjects [68]. Such changes could be considered a sign of nonspecific immune defense activation [69].

The third involved the production of antibodies. A vaccine against influenza was used in the study wherein some of the volunteers were restricted to 4 h of sleep per night before and after vaccination. It emerged that the number of antibodies in participants who kept regular rest was two times higher than the number of antibodies in those who suffered from SD [12]. The depletion of antibodies undoubtedly increases the risk of various infections. More importantly, the sleep effects in the study still presented at a 1-year follow-up, indicating that sleep helps maintain long-term antigenic memory [12].

The fourth was on the intestinal barrier of the host. The effects of SD on the intestinal barrier cannot be neglected. For example, studies have showed that the crucial factors of the intestinal barrier, like goblet cells, mucin 2 (MUC2), three tight junction proteins (Claudin, Occludin, ZO-1, TJs), and zonulin (one of the permeability indexes) all display signs of diminished function after SD treatment [54,55], thus causing the higher permeability of the barrier and chronic mucosal injury. Collectively, these findings suggest that sleep loss-induced immune dysfunction could be a significant factor contributing to a wide variety of disorders.

#### 3.1.2. The Potential Roles of Gut Microbiota in SD-Induced Immune Diseases

After presenting the deleterious effects of SD on the host immune system, it would be interesting to decipher how SD severely weakens our immune functioning. One recent study has yielded a deep insight into SD-treated gut microbiota and its experiment methods are very inspiring. First, the researchers assessed whether the absence of gut microbiota influenced inflammation after SD by comparing germ-free (GF) mice with specific pathogen-free (SPF) mice. Interestingly, in SPF mice, the number of pro-inflammatory cytokines (IL-1β, IL-6 and TNF-α) was significantly increased, while anti-inflammatory IL-10 was reduced. However, no differences in cytokines levels were discovered after SD in GF mice. Moreover, goblet cells, MUC2, and serum endotoxin levels were all decreased after SD in SPF mice, but GF mice showed no signs of intestinal barrier impairment. Such consequences demonstrated that the gut microbiota plays a mediator role in immune system weakening elicited by SD. Then, fecal samples from healthy and SD participants were randomly selected and transplanted into GF mice (divided into recipient baseline (rBL) and recipient SD (rSD) mice, respectively). The rSD mice exhibited higher levels of pro-inflammatory cytokines, serum endotoxin, lipopolysaccharides (LPS), lipopolysaccharide-binding protein (LBP), and Toll-like receptor 4 (TLR 4), along with stronger phosphorylated NF-κB (nuclear factor kappa-B), p65 expression, and more permeable colon mucosa, yet none of these were detected in the rBL mice. These findings indicate that GF mice transplanted with SD microbiota own more active TLR4/NF-κB inflammatory pathways and more permeable intestinal barriers [54]. Putting the two results together, it is effortless to realize that gut microbiota alterations play a crucial role in SD-induced immune diseases. More generally, gut microbiota participates in SD-induced immune diseases through the following pathways.

(1) Through the regulation of TLR4 and NF-κB gene expression.

As mentioned above, colonizing SD microbiota makes GF mice acquire a more activated TLR4/NF-κB inflammatory pathway. In this process, LPS, one of the most essential membrane substances of nearly all Gram-negative bacteria [70], is the expression activator. Working as a microorganism-associated molecular pattern, LPS, with LBP, is recognized by receptors on innate immune system cells such as TLR4 [71]. Along with activating TLR4 when confronting pathogens, LPS promotes the transcription of NF-κB inflammatory genes including TNF-α, IL-6, and IL-1β [54,72,73]. LPS and LBP have been confirmed to parallelly increase in acute infections and sepsis [74,75]. One of the most direct pieces of evidence for LPS being the crucial agent in this process is its increased levels in the serum of rSD mice, which has not been observed in the serum of rBL mice [26,54].

(2) Through the intestinal barrier.

The intestinal barrier is vital in the interactions between gut microbiota and the immune system. It performs the task of preventing various bacteria in the GI tract from entering into the blood. However, the research has further uncovered the deficient expression of intestinal colonic MUC2 and the reduced number of goblet cells in rSD mice. Such changes and the further damage of the intestinal barrier were not observed in rBL mice despite their defects in intestinal structure according to a previous study [76].

The intestinal barrier’s structure consists of three parts. The first is the outer mucus layer, constituted of mucus, commensal bacteria, antimicrobial proteins, and secretory immunoglobulin a, the second is the central single cell layer with specialized epithelial cells such as goblet cells, enteroendocrine cells, Paneth cells, and microfold cells, and the third, which comprises inner lamina propria and residing immune cells, acts as an immune barrier [77]. In and between intestinal epithelial cells (IEC), there are three types of junctional complexes acting as upholders and sealers of intercellular space: tight junctions (TJs), adherens junctions, and desmosomes [78].

Some species of commensal bacteria have been reported to participate in the normal physiological functions of intestinal barriers. For example, *Bacteroides* and *Faecalibacterium* can promote goblet cell differentiation, resulting in an increase of goblet cells and mucus gene expression [62]. In addition, an outer protein in *Akkermansia muciniphila* has the capability to initiate several signaling pathways including the upregulation of intercellular TJs [63]. A study explored the relationships between these microbiota and intestinal barrier indexes after SD, indicating that in line with causing the decreased abundance of *Akkermansia*, *Bacteroides*, and *Faecalibacterium*, SD causes a decline in goblet cell numbers, mucus production, and TJ expression [55]. Such results demonstrate the potential roles of SD gut microbiota dysbiosis in SD intestinal barrier impairment. Moreover, SCFAs produced by gut microbiota can also regulate the intestinal barrier by modulating mucin synthesis [79] and junction complexes [80] in normal cases. Consistently, it has been found that the concentrations of acetate, propionate, and butyrate experience significant decreases in SD mice [54].

(3) Through colonization resistance.

Colonization resistance (CR) is a term used to describe commensal microbiota’s ability to resist pathogenic bacteria invasion [21]. One of our previous studies has demonstrated that SD damages intestinal CR by disordering the pool of secondary bile acids with a decline of deoxycholic acid (DCA) [66]. Furthermore, we revealed that DCA, as the most abundant and important metabolite of mammalian secondary bile acids, could disrupt membrane permeability and exacerbate oxidative damage, thus reducing intestinal pathogen burden. We clarified that the abundance alterations of *Clostridium* spp. after SD may be involved in the decrease of CR in SD mice. Specifically, we suspected that Clostridium_UCG-014 was a beneficial population to support CR by producing efficient DCA. However, the specific biofunctions of Clostridium_UCG-014 remain to be explored.

The weakening of CR function means that the vulnerability of the body is totally exposed to exogenous pathogens. Therefore, one of the most prominent outcomes of SD is increased risk of infection. Exotic pathogens, especially multidrug-resistant (MDR) bacteria, which could have been prevented by host CR, can invade the intestine through the diet or other means. Over time, the intestinal lumen could eventually become a repository for antibiotic resistance genes (ARGs) [81]. Coupled with the impaired intestinal barrier, these pathogens break through the line of immune defense, pouring into blood, and release a variety of toxins or destructive enzymes. Such a pathological process often involves inflammation responses. Meanwhile, increased pro-inflammatory cytokines are also involved in the formation of adverse environments. Therefore, it becomes easier for SD-related infections to develop into chronic inflammation, which facilitates the development of IBD [1]. Moreover, it is worth mentioning that there is a bidirectional relationship between gut inflammation and dysbiosis: that is, inflammation can cause dysbiosis [82] and dysbiosis may lead to inflammation [83], creating a vicious cycle.

### 3.2. Metabolic Diseases

Metabolism is a general term for life-sustaining chemical reactions in the body whose functions cover three parts: energy conversions from food to cellular processes, energy supply for organic compounds of proteins, lipids, nucleic acids, and carbohydrates, and the removal of metabolic waste. A set of metabolic abnormalities, including hypertension, central obesity, insulin resistance, and atherogenic dyslipidemia, are collectively known as metabolic syndrome [84]. It is suggested that SD is linked with high risks of metabolic syndrome (i.e., obesity, T2DM, and high blood pressure), presenting a U-shaped relationship specifically [85,86], in which 7 h sleep is the optimal duration. Those sleeping for less than 7 h or more than 7 h are at higher risk than those sleeping for 7 h, and those sleeping 5 h or 9 h show similar risk [86].

Epidemiological studies have shown that SD is connected with excessive food intake and weight gain, directly pointing to obesity. The root cause of obesity is an excess of caloric intake over expenditure [87]. In respect of energy ingestion, appetite regulation is the center of attention that determines the quality and quantity of food absorption, leading to enormous intake when the balance breaks. Some researchers have offered deep insights into the effects of SD on appetite-regulating hormones including leptin, ghrelin, glucagon-like peptide 1(GLP-1), and peptide YY(PYY) [88]. Leptin is an adipocyte-derived hormone [89] that has the capability to regulate the balance between satiety and appetite via food intake [90]. Ghrelin, one kind of hormone produced by the stomach, is in synergy with leptin by stimulating appetite [91] and suppressing energy consumption [92]. Several studies have indicated the decreasing trend of leptin and an increase in ghrelin levels after SD [93,94]. Similar results have basically been verified in subsequent experiments [95,96]. Interestingly, gender-specific results have been found for the levels of GLP-1 (an intestinal-derived hormone), which elevate among women and decrease among men [88,97]. PYY, exerting anorexigenic effects, lessens after SD [88]. Moreover, the acute-phase protein serum amyloid A (SAA) is reported to be important in obesity. SAA owns a growth factor-like property and is able to attach to TLRs, enabling SAA to participate in inflammatory and metabolic processes. An investigation monitored the levels of SAA before and after SD and found that there was an increase in serum SAA in participants after two days of SD that could lead to obesity and insulin resistance [98]. SD also suppresses cognitive functions and activity in cortical brain regions, leading to higher calorie food selections [99]. One study investigated the relationships between sleep fragmentation (SF) and gut microbiota. GF mice receiving the gut microbiota of SF mice presented with an increase in food intake, and this was accompanied by the growth of highly fermentative members of Lachnospiraceae and Ruminococcaceae and a decrease of Lactobacillaceae families. These effects gave rise to white adipose tissue inflammation via intestinal barrier disruption [100]. However, since the experiment model mainly focused on SF, whether its conclusions still hold in SD must be confirmed.

Indeed, adiposis is connected with gut microbiota owing to the changes in composition and abundance in the latter. For instance, obese mice (leptin genetically engineered mice) have been found to have fewer Bacteroidetes, correlating with more Firmicutes, compared with lean mice [101,102], accompanied by the ratio of F:B rising. It has been proven that the increase of Firmicutes is likely related to the higher capability of transferring indigestible polysaccharides to monosaccharides and SCFAs, both in animals and humans [103]. It has also been shown that a 20% increment of Firmicutes with a corresponding Bacteroidetes decrease in humans could lead to the acquirement of 150 kilocalories [104]. Although we can confirm the essential role of gut microbiota in SD-mediated obesity, the specific mechanisms warrant more studies.

As for diabetes, it has been separated into two types including type 1 diabetes mellitus (T1DM) and type 2 diabetes mellitus (T2DM). Sleep has a more profound effect on the latter [58,105,106]. A large number of reports have verified that SD has the ability to cause alterations in glucose tolerance, acute insulin response to glucose, glucose effectiveness, and insulin sensitivity, all presenting a trend of decrease [30,58,106]. The mechanisms linking SD and abnormal glucose metabolism lie in several aspects: (1) the activation of the sympathetic nervous system can lower β-cells responses to glucose, thus resulting in reduced insulin sensitivity; (2) increasing free fatty acids brought by SD via promoting gluconeogenesis can lead to insulin resistance and hyperglycemia; and (3) the inflammation caused by SD, as mentioned above, can lead to insulin resistance, mainly via cytokines and corresponding signaling pathways (TNF-α & IKKβ/NF-𝜅B pathway, IL-6 & JAK-STAT pathway) [107,108]. Although there has been no direct evidence that microbiota dysbiosis is indispensable in SD-induced diabetes so far, there are great possibilities in view of the inseparable connections between T2DM and obesity.

The third metabolic syndrome related to SD is hypertension, referring to the abnormal rise of blood pressure. Hypertension is also considered as a contributing factor to cardiovascular diseases—the higher blood pressure levels are, the more risk the host has for other health problems such as heart disease, heart attack, and stroke. Data from British and American studies have suggested a consistent relationship between SD and the possibility of suffering from hypertension [109], which has been specifically shown as having a value of 21% in another report [110]. Besides, it has also been verified that SD not only raises night blood pressure, but also continues hypertension into the daytime [111]. Taken together, the available evidence suggests that SD can indeed trigger a range of metabolic-related diseases, but the underlying mechanisms remain to be further investigated, particularly those related to gut microbiota dysbiosis.

### 3.3. Gut-Brain Axis

In addition to several major systems, the brain, as the center of the human body, is the first organ to be affected by sleep. Evidence has proven that sleep plays a crucial role in brain functions, such as cognitive performance [2], memory consolidation [3,4], and mood regulation [5,6]. At the same time, the effects of sleep loss on the brain are profound and abominable. Studies have clarified that SD is closely related to neurological disorders, covering three major sectors. (1) Behavioral changes, memory and cognition decline: the disruption of the intracellular cyclic adenosine monophosphate (cAMP)-protein kinase A (PKA) signaling is associated with memory impairment, cognitive decline, and psychiatric illness [112]. (2) Alzheimer’s disease (AD): the typical features of AD include the deposition of extracellular amyloid β- (Aβ-) plaques, intracellular tangles, and neuronal loss. SD has been proved to increase Aβ- in the hippocampus, precuneus, thalamus, and cortex, along with neuroinflammation and oxidative stress, and inhibit cholinergic neurons, indicating a direct connection between SD and neuropathological events associated with AD [113]. (3) Stroke: a study showed that SD exacerbates stroke by raising the expression of growth-inhibiting genes, neuroinflammation, and oxidative stress [28,113].

The gut-brain axis plays a critical role in SD-induced neurological disorders. ‘Gut-brain axis’ refers to the bidirectional signaling between the GI tract and central nervous system (CNS) [114], i.e., the bidirectional communications between gut microbiota and brain. Most GI physiological activity is controlled by the enteric nervous system (ENS), i.e., the muscle sensorimotor and mucosa secretory connect the intestine to the spinal cord and the brain through primary afferent and autonomic fibers. Beyond the independent modulation of ENS, CNS can also regulate the intestine directly [115]. Meanwhile, the gut is responsible for sending information to the brain through complex pathways and signaling mechanisms to maintain microbiota-gut-brain (MGB) homeostasis. When such homeostasis is disrupted, the body may develop symptoms of eating disorders [116], autism spectrum disorders (ASD) [117], and, in particular, cognitive impairment.

To ensure the critical role of gut microbiota in the process of SD-induced cognitive impairment, researchers conducted an in-depth study. They chose SPF mice and GF mice and subjected them to the same level of SD. It turned out that the recognition index (RI, a measure of recognition memory that refers to the relationship between the time spent on investigations of novel objects and total object investigation, namely [RI = T_N_/(T_N_ + T_F_)] (T_F_, familiar objects, T_N_: novel objects) [118]) was decreased by 24.26% in SPF SD mice, but no changes were observed in GF SD mice. Subsequently, gut microbiota from the fecal samples of 40 h SD humans was transplanted into GF mice. Interestingly, the GF recipient mice exhibited neuroinflammation phenotypes in the dorsal hippocampus (dHPC) and medial prefrontal cortex (mPFC), with increasing levels of Iba1-positive cells (including microglia [119]) and pro-inflammatory cytokines such as IL-1β, IL-6, and blood-brain barrier (BBB) permeability marker S100β. These results revealed that SD-triggered gut microbiota dysbiosis is a crucial factor in initiating neuroinflammation [54].

The main reason for this is the increased permeability of BBB, which allows harmful metabolites to enter the brain and impair neuronal functions. Microglia are the resident immune cells of the brain, and their increase indicates the currency of neuroinflammation [120]. Neuroinflammation is a term used to describe the activation of resident immune cells in CNS and is associated with many cognitive disorders. Some studies have reported that SCFAs can inhibit neuroinflammation and are involved in regulating microglial functions. Among SCFAs, butyrate has the most significant effects. Its circulating levels have been found to be negatively correlated with cognitive impairment and neuroinflammation [121]. Collectively, gut microbiota exhibits irreplaceable effects in the neurological dysfunctions caused by SD, whereas the more specific mechanisms remain to be explored.

### 3.4. Other Sleep-Induced Diseases

Besides the disorders mentioned above, SD can also result in high risks of cardiovascular [122], respiratory [123], musculoskeletal [124], and nephrology [125] dysfunctions. However, given that these diseases are inconsistent with the topic of this review, they will not be presented here. Related content can be consulted in another review written by Liew et al. [112].

## 4. Conclusions and Implications

Because of the considerable roles of SD in negatively affecting human health, it is urgently needed to develop effective strategies to tackle this issue. Therapies to translate research into medical interventions are under active investigation. Therefore, we provide several potential countermeasures here from two different perspectives.

The first is to ensure adequate sleep and improve the quality of sleep, which can address SD-related concerns from the source. Melatonin (*N*-acetyl-5-methoxytryptamine), a kind of neurohormone produced by the pineal gland and possibly all extrapineal organs, is able to transmit information about darkness and contributes to the synchronization of circadian oscillators [126]. Accordingly, melatonin has been widely applied as a sleep-promoting agent by inhibiting orexin neurons in the hypothalamus. Other versatile physiological and pharmacological biofunctions of melatonin have also been uncovered, including immunomodulation and the inhibition of cellular apoptosis. It has been shown that melatonin, a hormone that plays a key role in maintaining the circadian rhythm, can effectively reverse harmful SD-induced effects [55,119]. In one study, with supplements of melatonin, experimental mice’s α-diversity, ACE, Chao, and Shannon indexes, which initially reduced, all presented an increasing trend, reaching the same levels as in the control groups. As for the specific alterations of gut microbiota, supplementation with melatonin to SD mice induced an increase in *Akkermansia*, *Bacteroides*, and *Faecalibacterium* and a fall in *Aeromonas* [119]. Consistently, the F:B ratio also came back to normal. Moreover, melatonin treatment can also counteract the negative effects induced by SD, covering pro-inflammatory reactions, gut microbiota dysbiosis, activation of the TLR4/NF-κB pathway, and intestinal barrier dysfunction [55]. Therefore, we might use melatonin as a beneficial agent for the treatment of related diseases caused by SD. Besides melatonin, there is another drug that is easy to mention when it comes to SD: caffeine. It has been confirmed that caffeine has the functions of improving reaction time and physical performance after SD [127,128]. Moreover, SD mice treated with caffeine presented a reverse in Verrucomicrobia and Proteobacteria, along with a decrease in Firmicutes and Bacteroidetes, compared with normal-sleep mice [129]. Caffeine itself processes antioxidant properties [130] and anti-inflammatory activity by inhibiting the secretion of inflammatory cytokines [131]. However, it must be mentioned that caffeine cannot replace sleep and that it actually will exacerbate SD [132]. Meanwhile, butyrate supplementation has also been proven to enhance sleep [31,133]. Butyrate, a four-carbon SCFA, is a product of the intestinal microbial fermentation of indigestible foods. The absorption of SCFAs starts from the intestines and proceeds into the portal circulations, and then they directly arrive at the liver. Butyrate transmits signals from the free fatty receptor 2/3 (FFAR2/3), with both of them being expressed in the liver, and FFAR3 is also expressed by the portal vein wall. Through these receptors, the sleep-promoting effects of butyrate are regulated by a sensory mechanism of the liver and/or portal vein wall. Moreover, butyrate has powerful anti-inflammatory properties, being able to suppress the production of pro-inflammatory cytokines and the activation of NF-κB expression, thus inhibiting colonic and liver inflammations [133]. Therefore, the potential beneficial effects of butyrate on SD deserve more attention.

Considering the indispensable role of gut microbiota in SD-related diseases, targeting gut microbiota dysbiosis may serve as a distinct strategy to relieve symptoms associated with SD. As mentioned above, CR was remarkably impaired after SD with DCA imbalance. Our study also brought forward treatment with nicotinamide mononucleotide (NMN), a product of the nicotinamide phosphoribosyl transferase reactions, to reverse such adverse situations. Our results demonstrated that NMN supplementation restored intestinal CR by modulating intestinal microbiota and remodeling the metabolism of secondary bile acids. Specifically, NMN supplementation increased the levels of DCA in the gut, which exhibited excellent antibacterial activity and synergistic activity in combination with existing antibiotics against pathogens. Furthermore, in their daily routine, people with SD gut microbiota disorders can intentionally consume probiotics [134] and prebiotics [135]. Probiotics have been confirmed to have the function of preventing the growth of pathogens by increasing the production of β-defensin and IgA. They can also strengthen the intestinal barrier by maintaining TJs and inducing mucin synthesis [136]. As for the effects of probiotics on gut microbiota compositions, however, no study clearly demonstrates the direct relationships between altering gut microbiota compositions and probiotics treatment [136]. However, one investigation proved that the beneficial effect of probiotics was associated with the stabilization of intestinal microbiota [137]. Prebiotics, which are compounds in food supporting beneficial microorganisms’ growth and activity, can promote the production of SCFAs, presenting synergistic effects. A recent narrative review evaluated the potential of pro-, pre- and postbiotic treatments in improving sleep quality and ameliorating stress and anxiety [138].

**Figure 3:**
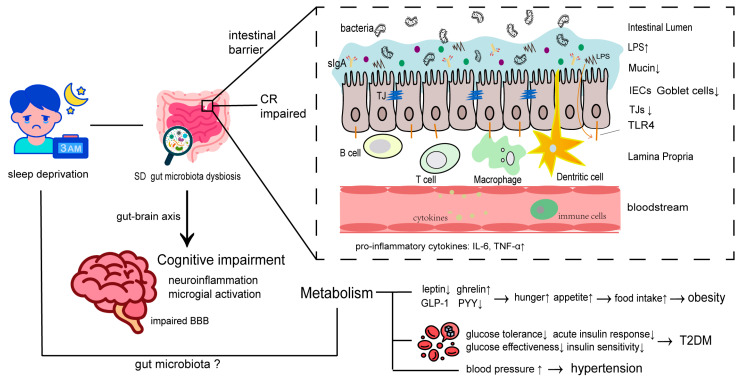
Summary of SD-induced diseases and their relationships with SD-induced gut microbiota dysbiosis. In the immune system, SD gut microbiota disorders lead to broken intestinal barriers and impaired colonization resistance, which can manifest in decreased mucin, IECs goblet cells, TJs, and anti-inflammatory cytokines, as well as increased LPS and pro-inflammatory cytokines. In the metabolic system, SD contributes to obesity, T2DM, and hypertension; the effects of gut microbiota in this process have been fully characterized. In the nervous system, SD gut microbiota disorders result in decreasing functions of cognition via neuroinflammation and microglial activation. The up/down arrows indicate the increase/decrease of the represented object.

In conclusion, these findings demonstrate that SD causes gut microbiota dysbiosis, and that such disorders lead to systemic changes in the whole body including weakened immune defense, increased energy intake, broken glucose metabolism, and impaired cognitive functions (Figure 3). However, most studies clarify their own or pairwise mechanisms and consequences of SD, gut microbiota dysbiosis, and gut microbiota-related diseases rather than clarifying the connections among these three. Besides, gut microbiota-derived metabolites deserve more priority in future studies since their functions spread throughout the body. Taking SCFAs as an example, here we list several of their effects that have not been mentioned above. (1) SCFAs can be absorbed by intestinal epithelia cells as nutrient supply and contribute to a lower intestinal permeability, (2) SCFAs promote mucus secretion experiences by elevating the PGE 1 & 2 ratio, (3) SCFAs act on FFAR2 and FFAR3 and transmit signals to peripheral and CNS areas, thus inducing intestinal gluconeogenesis [139], and (4) intracellular butyrate, propionate, and acetate work as histone deacetylase inhibitors, promoting gene transcription and favorable treatment for neuropsychiatric diseases like enhancing cognitive function in fear [140]. More importantly, the levels of SCFAs encounter significant changes after SD. These SCFAs include butyrate, acetate and propionate, whose numbers all present a decreasing trend with extension of SD duration.

Besides, we raised two issues, focusing on the topic of our review, that still need to be resolved:

1. Most of the results so far have been obtained in animal models, and there is still a lack of human clinical data to support these findings. Moreover, the time periods for SD treatment have been too concentrated. Such experiment designs tend to be ideal and not in line with the actual situation.
2. It is not known what the detailed mechanisms behind the disruption of gut microbiota (caused by SD) are. Whether altered intestinal environments also play an important role in this process is unknown. In addition, there is a lack of enough evidence to explain how gut microbiota affects the development of human diseases in the context of SD.

## Figures and Tables

**Table 1:** Summary of SD experiments. This table gathers information about experiment subjects, protocols and results from four representative studies. PSD: partial sleep deprivation, F:B ratio: Firmicutes: Bacteroidetes ratio. The up/down arrows indicate an increase/decrease of the corresponding bacteria.

Subjects	Experiment Protocols	Results	Ref.
C57BL/6 mice	repeated SD 20 h SD/day for 5 days	F:B ratio↑ *g_Lactobacillus*↓ *g_Bifidobacterium*↓ phylum Actinobacteria↓	[57]
Nine normal-weight men	two nights of PSD; sleep opportunity 02:45–07:00 h	F:B ratio↑ families Coriobacteriaceae and Erysipelotrichaceae↑ phylum Tenericutes↓	[58]
Twenty-five healthy participants (13 males)	40 h of SD	α-diversity: 24 h SD↓, 40 h SD↓↓ β-diversity obvious different *g_Prevotella*↓ *g_Sutterella*↓ *g_Parasutterella*↓ *g_Alloprevotella*↓ *g_Anaeroplasma*↓ *g_Elusimicrobium*↓	[54]
CD1 mice (male)	Continuous SD for three days	α-diversity↓ ACE, Chao and Shannon indexes↓ Simpson index↑ phylum Bacteroidetes↓ phylum Firmicutes↑ F:B ratio↑	[55]

## Data Availability

No new data were created or analyzed in this study. Data sharing is not applicable to this article.
